# Genome-Based Studies of Marine Microorganisms to Maximize the Diversity of Natural Products Discovery for Medical Treatments

**DOI:** 10.1155/2011/384572

**Published:** 2011-08-02

**Authors:** Xin-Qing Zhao

**Affiliations:** School of Life Science and Biotechnology, Dalian University of Technology, Linggong Road 2, Dalian 116024, China

## Abstract

Marine microorganisms are rich source for natural products which play important roles in pharmaceutical industry. Over the past decade, genome-based studies of marine microorganisms have unveiled the tremendous diversity of the producers of natural products and also contributed to the efficiency of harness the strain diversity and chemical diversity, as well as the genetic diversity of marine microorganisms for the rapid discovery and generation of new natural products. In the meantime, genomic information retrieved from marine symbiotic microorganisms can also be employed for the discovery of new medical molecules from yet-unculturable microorganisms. In this paper, the recent progress in the genomic research of marine microorganisms is reviewed; new tools of genome mining as well as the advance in the activation of orphan pathways and metagenomic studies are summarized. Genome-based research of marine microorganisms will maximize the biodiscovery process and solve the problems of supply and sustainability of drug molecules for medical treatments.

## 1. Introduction

**Figure 1:**
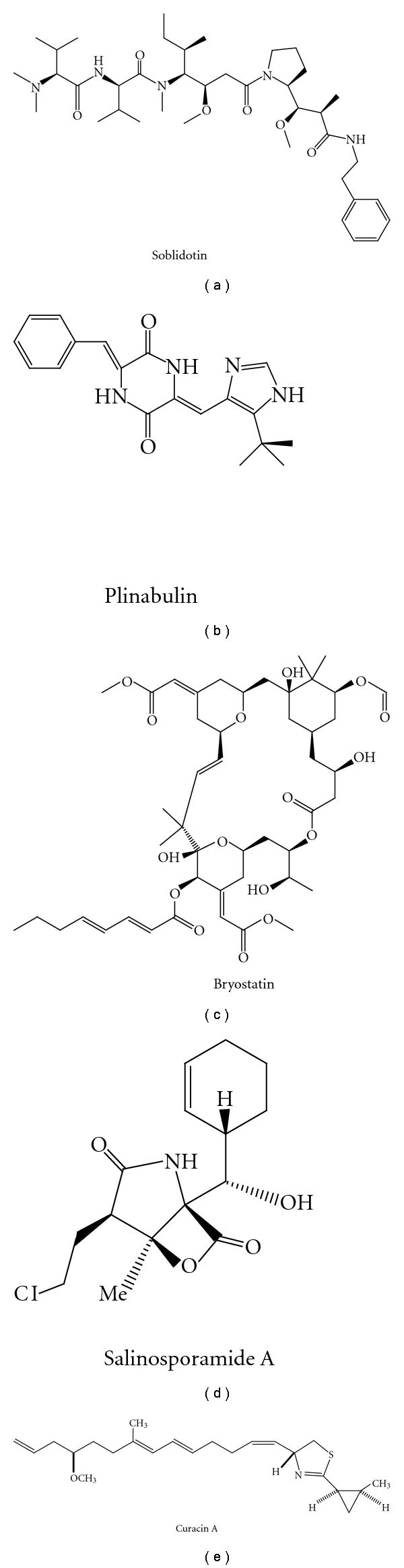
Structures of the marine microbial-derived compounds in clinical development.

The marine environment covers more than 70% of the Earth's surface and has been proven to be a rich source for both biological and chemical diversity. Marine natural products have become fascinating targets for biologists and chemists for discovery of lead compounds for clinical development for the past five decades [1–10]. Marine microorganisms comprise an important group of natural product producers, and the natural products isolated from marine microorganisms presented diverse activities, such as antibacterial, antifungal, anticancer, and antiviral activities. Of the major producers of useful natural products, marine actinobacteria are especially notable for the capability of producing diverse useful natural products [5–7], and marine cyanobacteria are also an important group of bioactive metabolites [5]. Currently 13 marine-derived compounds are reported in clinical development, and those belong to the authentic or derivatives of marine natural products of microbial origin and are listed in Table 1, and the structures are displayed in Figure 1. Of these compounds, soblidotin (TZT 1027), the synthetic derivative of dolastatin 10, was isolated from the marine cyanobacterium *Symploca *sp. VP642 from Palau, and the synthesis of plinabulin (NPI-2358) was inspired by halimide and phenylahistin, the former of which was isolated from a marine fungus *Aspergillus* sp. CNC-139. The structures of other interesting compounds with novel structures or potent activities can be retrieved from the recent literatures and review papers [8–17]. 

Although natural products embrace a wide range of entities such as biomaterials and biocatalysts as well as biocontrol agents other than drug molecules, due to space limitation, in this paper, most of the cited natural products focused upon are compounds with the potential to be developed as drugs and used in medical treatments. Taking the advantage of established microbial cultivation technology, the promising natural products identified from marine microorganisms provide great potential to solve the supply and sustainability problem of useful drugs to cure human diseases. 

High-throughput screening (HTS) of drug molecules demands abundant diversity of the compound libraries to get more hits for drug discovery. To combat the emergence of drug-resistant pathogens, as well as to improve the efficacy and safety of medical treatment, new drug leads are urgently needed. The key point of improving the success of natural product screening and development is to improve the diversity and the size of natural product library for new drug discovery. In this paper, the recent progress in the genomic research for natural compound discovery is summarized, with the focus on the important roles of genetic diversity deduced from genome mining on new drug discovery. 

## 2. Genomic Sequencing of Natural-Product- Producing Microorganisms

Genomic sequencing of microorganisms in the recent years has unveiled unprecedented insights into the biosynthetic potential of microbial cells, and thus the discovery of natural products has entered into the new postgenomic era. The genomic sequencing data of various living organisms have been accumulating rapidly in recent years. According to the Genomes On Line Database (GOLD), there are 5831 genome sequencing projects registered in GOLD, while according to the last update (April 11, 2011), 10298 genome projects are documented currently in GOLD (http://www.genomesonline.org), of which 1674 complete genomes are registered. And in the mean time, 204 archaeal, 6087 bacterial, and 2003 eukaryal genome projects are ongoing, which are double of those reported in 2009 [18]. The same case is the genome project numbers in 2009 comparing with those in 2007 [19]. Genome sequencing projects supported by the Gordon and Betty Moore Foundation resulted in the release of about 150 marine bacterial genomes [20]. Draft or complete genomic sequences of more than 35 marine cyanobacterial strains of six different species have been released, and some gene clusters for polyketide and nonribosomal peptide biosynthesis were identified [21]. It can be anticipated that more marine microbial genomic sequences will be available in the near future, and more relevance of natural product biosynthesis to the microbial genomes will be disclosed. For instance, the genomes of marine *Nocardiopsis* sp. strain TFS65-07 and marine-derived fungus strain *Aspergillus* sp. MF297-2 were reported to be sequenced, and studies on the biosynthesis of thiopeptide antibiotic TP-1161 and prenylated indole alkaloids, stephacidin and notoamide metabolites, were subsequently guided by the bioinformatics analysis of gene clusters located in these genomes [22, 23]. Despite the fact that comparing to the studies on terrestrial species and strains, genomic studies of marine-derived microorganisms are still well performed, the knowledge accumulated from the terrestrial microorganisms can be used also in the marine-derived strains. Actinobacteria are major producers of various useful antibiotics and antitumor agents. In the below section, I gave some examples on the investigations on the genomes of some model or natural-product-producing strains, which will shed light on their marine-derived counterparts.

*Streptomyces* is the largest genus of actinobacteria, and, species of streptomycetes are major producers of antibiotics which have wide range of utilities in medical treatments and agricultural applications. The first genomic sequencing of the model streptomycete, *Streptomyces coelicolor* A3 [2], was reported in 2002 [24], and the genome contains about 31 biosynthetic gene clusters that were predicted to contribute to the biosynthesis of secondary metabolites, which are the major source of bioactive natural products. Surprisingly, only half a dozen of these secondary metabolites in *S. coelicolor* have by that time been identified, which implies that microbial cells can produce much larger chemical diversity than previously anticipated. Later on, the genome sequence of *S. avermitilis *was also reported [25], followed by the blooming uncover of genomic sequences of various antibiotic producers [26–31]. 

The genome sequence of the first seawater-requiring marine actinomycete, *Salinispora tropica, *was reported [26]. Bioinformatics analysis revealed that this marine bacterial species owns a large proportion of genes (about 9.9%) responsible for natural product biosynthesis, while the corresponding numbers in *S. coelicolor *and *S. avermitilis* are 4.5% and 6%, respectively [32]. The genome of *S. tropica* contains amazingly large size of genes (516 Kb) dedicated to polyketide synthase (PKS) and/or nonribosomal peptide synthetase (NRPS) biosynthesis, which are megasynthases that are responsible for many active natural product biosynthesis and are especially popular tools for combinational biosynthesis [33–35]. The majority of the gene loci are novel, indicating that* S. tropica* has great potential to produce novel natural products and that the biosynthetic potential of this species, like that of many other actinomycetes that were genomically sequenced, is considerably greater than that has been observed by cultivation and chemical analysis [26], which provided basis for mining of these novel genetic sources for new natural products.

## 3. Important Roles of Genetic Studies to Maximize the Diversity for Natural Product Discovery

Although natural products from marine microorganisms have already widened the spectrum of chemical diversity, increasing the diversity of natural compounds is still the prerequisite for HTS to get enough new hits for drug discovery. And in the meantime, the classical activity-based screening is increasingly challenged by repeated discovery of known compounds, as well as the limitation of available assay methods. Therefore, new techniques are needed to maximize the diversity of natural products for the discovery of new therapeutic agents, not only to increase the efficacy and decrease the toxicity, but also to combat the emergence of drug resistance. 

**Figure 2:**
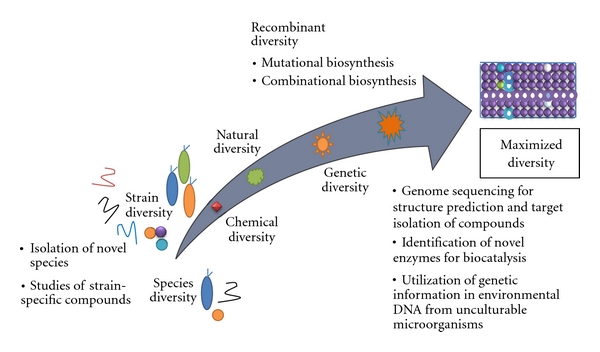
Important tools to maximize the diversity for new drug discovery.

The development of genomic sequencing techniques as well as the bioinformatics tools in recent years has profound influence on the discovery of new drugs. Nowadays it is much easier to have the genome of the interested organisms sequenced, especially for relatively small genomes of microorganisms. The availability of genomic sequences and the subsequent genome mining of microorganisms have provided new insights in the biosynthetic potential of the cells, and thus the studies of marine natural products have entered into the new postgenomic era. There is an increasing trend to expand the studies on the chemical diversity of natural products from bioassay-based screening to genetic diversity studies, from the studies of natural diversity to creation and utilization of recombinant diversity (e.g., to use genetic tools to produce new “unnatural” natural products), and in the meantime, it is well accepted that the chemical diversity and genetic diversity not only exist in different microbial species, but also exist in the different strains in one species [36]. Therefore, the chemical diversity for useful natural products can be maximized by the integration of genomic research and genetic engineering with the efforts of chemists. The important strategies to maximize biodiversity of marine natural products are illustrated in Figure 2, and will be depicted in detail in the following sections. 

## 4. “Gene-to-Compound” Procedure for Genome-Based Screening of Natural Products

It has been observed that the genetic elements responsible for the biosynthesis of many, if not all, secondary metabolites are clustered in the bacterial or fungal genomes to form gene clusters, which include the genes encoding biosynthetic enzymes, as well as genes responsible for regulation and resistance [37]. This genetic feature has not only greatly facilitated the molecular cloning and characterization of the biosynthetic genes, but has also provided basis for the generation of novel compounds through combinational biosynthesis. Traditionally, to identify and isolate the gene cluster for natural product biosynthesis, knowledge on the chemical structures of the compounds must be obtained prior to the genetic studies. However, this “compound-to-gene” route is facing challenges in that (1) the cultivation media and conditions have great impact on the searching of the chemicals; therefore, a lot of efforts have to be made to test different media and cultivation parameters; (2) the bioassay-based screening results in the rediscovery of known compounds from the screening of natural products; (3) the elucidation of complex structures; (4) the isolation of the natural products is hampered by low production yields and complex purification procedures. 

In the recent years, more and more studies have focused on the “from genetics-to-chemistry” route for natural product research, that is, to use gene-based screening strategy to study the biosynthetic potential of the microbial strains, followed by molecular cloning experiments and chemical purifications [38, 39]. A good example is the studies on the identification of active halogenated compounds from 550 randomly selected actinomycetes based on the conserved sequences from diverse halogenase genes. Using this “genome-based” strategy, novel halogenase genes were identified and novel halometabolites were isolated by the cloning of related gene cluster in heterologous host [38]. We used the same strategy to screen the marine actinobacteria strain library in our lab and isolated a gene cluster putatively involved in the biosynthesis of glycopeptide antibiotics. Furthermore, the genomic sequence of a new marine-derived *Streptomyces* sp. S187 was obtained, and bioinformatics analysis guided the identification of the gene cluster responsible for a new glycopeptide antibiotic (unpublished data). Genome scanning is another successful approach, in which the random genome sequence tags (GSTs) prepared from the genomic DNA library are screened using degenerate primers to identify the gene cluster, and the products of the gene cluster can be subsequently searched using various genomics-guided strategies [40, 41], which will be detailed in the following section. Using this “gene-to-compound” strategy, screening of a large strain library can be rapidly focused on small group of strains with high possibility to produce new compounds. However, the success of this strategy is dependent on multifaceted factors, including the selection of target genes for genome-based screening, the design of suitable degenerate primers, the quality of template DNA (especially environmental DNA), PCR conditions, and sequence degeneracy, and thus is limited to the detection of chemical diversities with diverse structures.

## 5. Genome Mining of Microbial Natural Product Producers

Although the cost of genome sequencing is declining, it is still not affordable for every lab for natural product research. Two questions may arise concerning the whole genomic sequencing. Why do we need to know all the sequences on the genome? What can we do next after obtaining the genomic sequence? 

To answer the first question, it is well accepted that the genetic loci in the genome can be roughly grouped by genes involved in primary metabolism and genes involved in secondary metabolism. Because not all genes are expressed in all the conditions, the chemical analysis of the cultivation broth under certain conditions cannot fully explore the biosynthetic potential of the cells. Furthermore, not all genes can be fished out by gene-based screening using degenerate PCR; therefore, sequencing the genome can fully access the genetic loci responsible for natural product biosynthesis. At the same time, genetic loci involved in primary metabolism are also tightly linked to the biosynthesis of natural product by providing precursors and cofactors [42]; therefore, the production of natural products requires the balanced interaction of metabolic network of both primary metabolism and secondary metabolism. Knowing the genomic sequence is the prerequisite to understand and harness the diversity of the natural product producers. The availability of genomic sequence also enables the studies of natural product producer using systems biological approaches, which are exemplified by the metabolome studies [43] and transcriptome studies [44] in *S. coelicolor*. 

The answer to the second question relies on the rapid development of tools for genome mining, which were presented in several excellent reviews elsewhere [45–51]. Several strategies for genome mining for novel natural products identification are summarized below with the addition of recent reports:

**(1) Genome-Guided Structural Prediction of the Products and Target Isolation.**

As indicated above, the polyketides and nonribosomal peptides are synthesized by modular PKS or NRPS system, and extensive knowledge has been accumulated on the relationship between the structures of these compounds and the organization of the multienzymes. Successful mining of ribosomally synthesized peptides has also been reported and summarized [51]. Bioinformatics analysis of these groups of genes thus can give important insight into the structural features of the biosynthetic products. The genome-guided discovery of salinilactam A in marine actinomycete *S. tropica* strain CNB-440 is one good example [27]. Initial bioinformatises analysis indicated that the *slm* gene cluster codes for polyene macrolactam polyketide, and investigation of the fermentation broth using characteristic UV chromophores of polyene units led to the identification of structural fragments of salinilactam A series compounds. Inspection of the structure fragments suggested that salinilactam A was derived from a PKS with at least 10 extension modules. This information was useful to help resolve and properly assemble the repetitive DNA sequences associated with the *slm *PKS, which was used in combination of partial NMR-based structural fragments to accurately predict the gross chemical structure of salinilactam A. What is more, bioinformatics analysis also assisted in the correction of the initial assignment of the C-28 methyl group complicated by overlapping olefinic NMR signals. The salinilactam A structure was later verified by comprehensive NMR analyses of the purified natural product to confirm the bioinformatics-based total structure assignment.

In another recent report, salinosporamide K was discovered by comparative genomic analysis of “*S. pacifica*” with that of *S. tropica* [52]. A truncated biosynthetic gene cluster was identified in the draft genome of “*S. pacifica*”, which is related to the 41 kb gene cluster in *S. tropica* for salinosporamide A biosynthesis, but the genes coding for the enzymes in the chloroethylmalonyl-CoA pathway are absent in “*S. pacifica.*” This information guided the isolation of salinosporamide K, which structurally resembles salinosporamide A [26].

**Figure 3:**
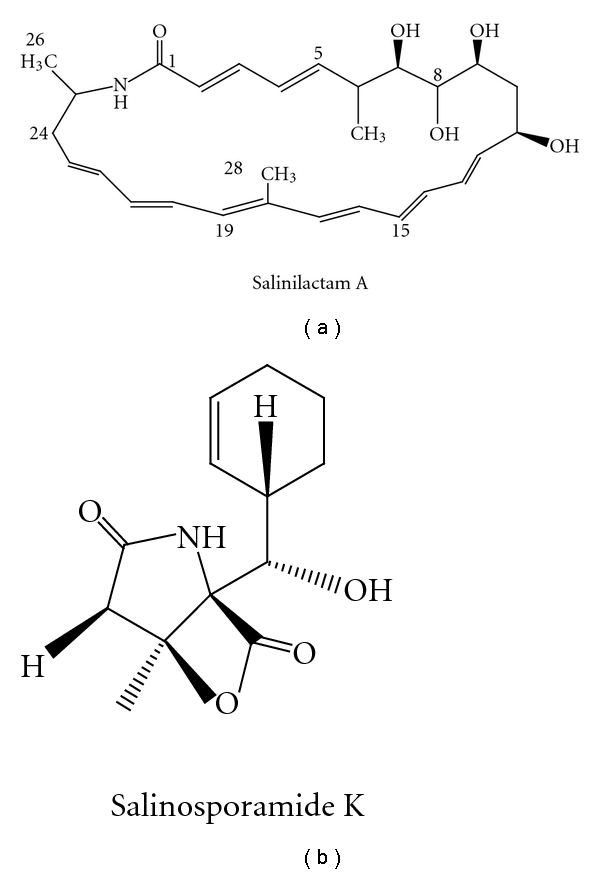
Structures of salinilactam A and salinosporamide K discovered by genome mining.

The structures of salinilactam A and salinosporamide K described above were presented in Figure 3.

**(2) Comparative Metabolic Profiling of the Mutants with the Addition or Inactivation of the Biosynthetic Genes.**

**Figure 4:**
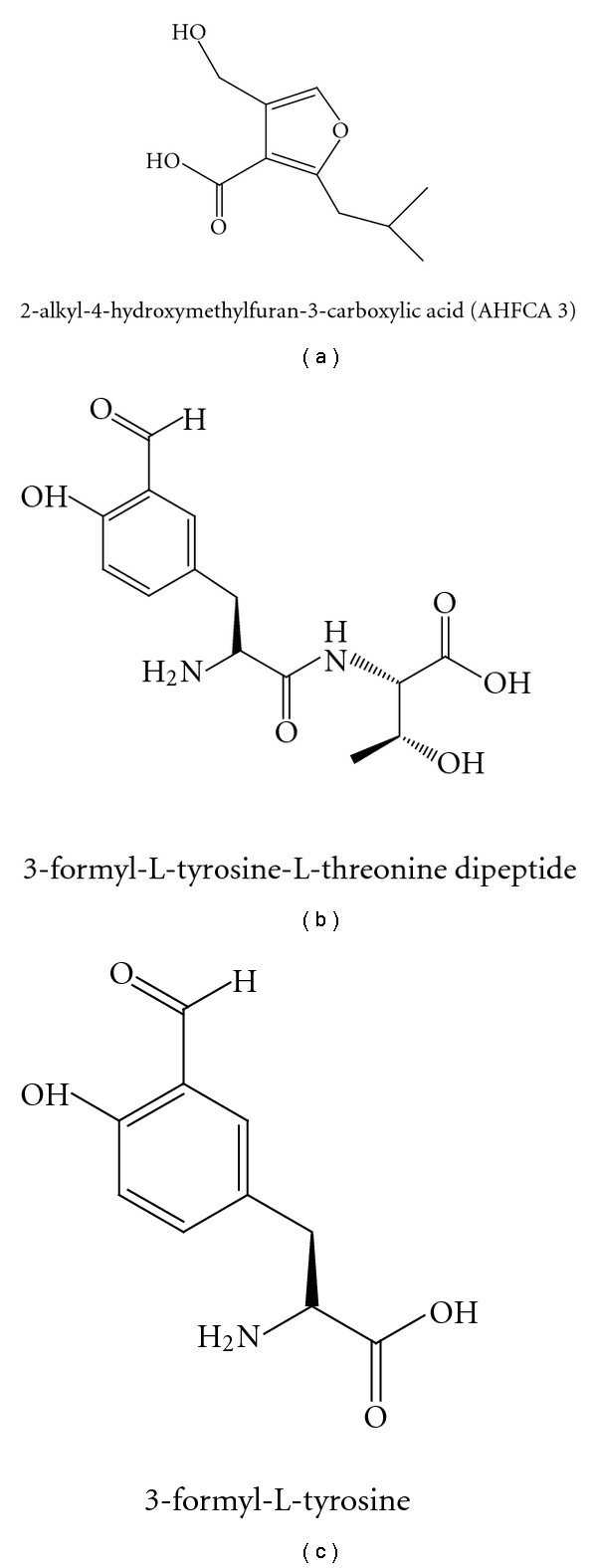
Structures of MMF1, 3-formyl-L-tyrosine-L-threonine dipeptide and 3-formyl-L-tyrosine.

Biosynthetic gene clusters that are related to the production of yet-unknown metabolites are often referred to as “orphan” [46]. The biosynthetic genes in the orphan gene cluster can be inactivated by deletion or disruption, and the comparison of the metabolic profiling using HPLC or LC-MS of the mutant with that of the wild type strain will identify the product of the gene cluster. The discovery of coelichelin in *S. coelicolor* is the early example in which this approach was employed [53]. Alternatively, the biosynthetic gene clusters can be heterologously expressed in a well-characterized “clean” host with no background production of similar metabolites, and the comparative metabolic profiling will help to identify the novel products. The 5 new 2-alkyl-4-hydroxymethylfuran-3-carboxylic acids termed as Mm furans (MMFs), which are new autoinducers to regulate antibiotic production, were discovered by this heterologous expression approach [54]. In a recent report, a gene cluster containing ATP-grasp enzymes identified by genome mining of the marine gamma proteobacterium *Pseudoalteromonas tunicata* D2 was overexpressed in *E. coli*, which led to the isolation of two new metabolites, 3-formyl-L-tyrosine-L-threonine dipeptide and 3-formyl-L-tyrosine [55]. This work emphasized the studies of smaller pathways (comparing to PKS and NRPS) employing less familiar biosynthetic strategies for the discovery of novel natural products. The structures of one Mm furans (MMF1), 3-formyl-L-tyrosine-L-threonine dipeptide, and 3-formyl-L-tyrosine were shown in Figure 4.

**(3) Activation of the Biosynthetic Genes by Manipulation of Regulatory Genes.**

The failure of producing certain natural products can be attributed to the poor expression or silence of the biosynthetic gene cluster. Therefore, overexpression of the regulatory genes, either pathway-specific regulatory genes or global regulatory genes, can lead to the production of novel compounds. One example is the discovery of aspyridones in *Aspergillus nidulans*. Overexpression of the putative pathway-specific regulatory gene located in the silent gene cluster resulted in the identification of the novel hybrid PKS-NRPS product aspyridone [56]. Alternatively, the promoter of the pathway-specific regulatory gene can be replaced by strong constitutive promoter, so that the expression of regulatory gene can be deregulated by repression.

On the other hand, some regulatory genes can also exert inhibitory effect on the production of certain compounds; in this case, the deletion of such regulatory genes readily results in the activation of silent pathways and production of novel compounds. In a recent report, a new yellow-pigmented secondary metabolite was observed in *S. coelicolor* after deleting a putative pathway-specific regulatory gene (*scbR2*) that encodes gamma-butyrolactone receptor protein [57]. This strategy can be used for the discovery of novel natural products from marine microorganisms, although no known literature in this aspect has been reported so far.

Other strategies for genome mining including genomisotopic studies and in vitro reconstitution of biosynthetic enzymes were described in reviews elsewhere [58, 59]. It can be predicted that genome mining of marine microorganisms will unveil the tremendous diversity of natural products produced by microorganisms. What is more, it should be mentioned that only 1% of the microbial community is estimated to be culturable in lab conditions [60–62], pointing out the importance the area of biodiversity research in the yet “unculturable” microorganisms, which will be addressed in the following section.

## 6. Metagenomic Studies of “Unculturable” Marine Microorganisms

Marine symbiotic microorganisms are rich source of active natural products and are thought in some cases to be the true producers of potent drug candidates instead of the marine invertebrate including sponges, tunicates, and bryozoans [63]. Shotgun sequencing of the environmental genome in the seawater samples collected from the Sargasso Sea near Bermuda also revealed tremendous diversity of the commonly thought tobe nutrient-limited environment [64], which unveiled 148 previously unknown bacterial phylotypes and 1.2 million previously unknown genes. However, the majority of the marine symbionts and 99% of the microbial population in environment are difficult to grow or are slowly growing as pure culture. To fully access the valuable chemical diversity and explore the biosynthetic potential of all the microbial communities, the environmental DNA (eDNA) is extracted directly from a given environment, and packed into vectors, following by transformation to various hosts, including *E. coli* and *Streptomyces lividans*. Clone libraries are produced independently of the host cultivation or fermentation and then subjected to sequence-based screening or function-based screening. This cultivation-independent approach is termed metagenomics. During the past decade, many successful metagenomic studies have been reported. Onnamides and theopederins are antitumor polyketides produced by an uncultured *Pseudomonas* sp. symbiont of marine sponge *Theonella swinhoei*. Biosynthesis genes of onnamides and theopederins were isolated from the metagenome of the marine sponge, and bioinformatics analysis of the biosynthetic genes strongly indicated a prokaryotic origin, suggesting that these potent antitumor agents may be produced by the symbiotic bacteria [65]. An entire putative gene cluster responsible for the biosynthesis of bryostatin, which has been evaluated for the treatment of various leukemias, ovarian cancers, and prostate cancers, has been implicated to be produced by a symbiotic bacteria, *Candidatus Endobugula sertula* from the marine bryozoan [66]. In another study, gene cluster for the biosynthesis of potent antitumor agent psymberin was isolated from the metagenomic library of marine sponge *Psammocinia* aff. *Bulbosa* [67]. Combining with the research development in heterologous expression [68], active compounds can be produced in large quantities by overexpressing the biosynthetic gene clusters isolated from metagenomic libraries in the heterologous hosts, which will solve the supply and sustainability problem of the precious compounds produced by marine symbionts and the yet “unculturable” marine microorganisms. 

**Figure 5:**
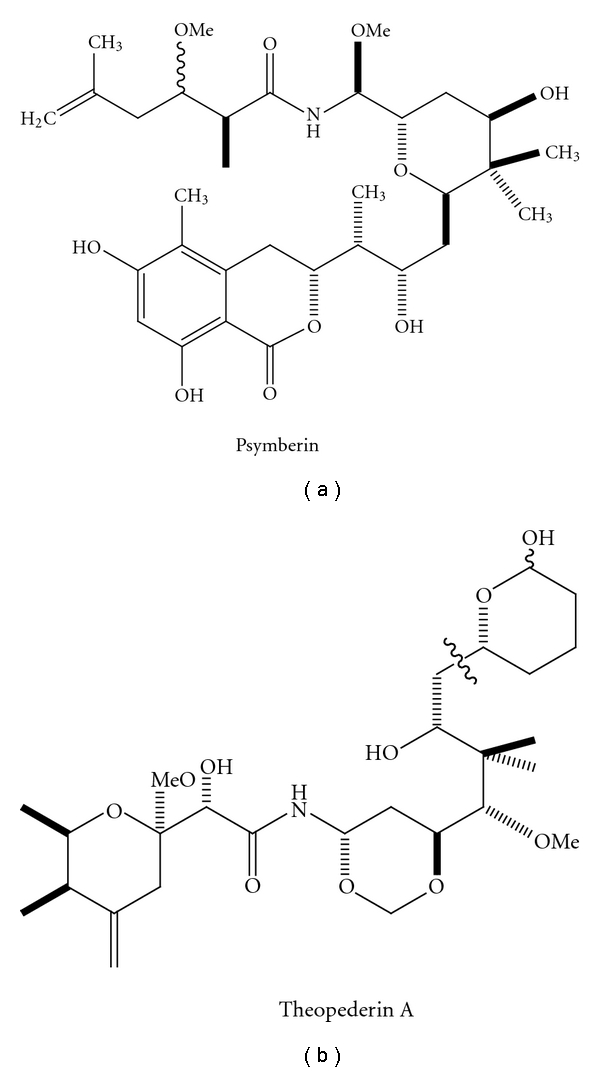
Structures of antitumor agents psymberin and theopederins A studied in metagenomic analysis.

The structures of some compounds studied by metagenomic analysis were presented in Figure 5.

## 7. Updating Tools for Exploration of Diversities of Natural Compounds

Exploration of the diversity of marine natural products is greatly promoted by various genome-based strategies described in this paper, and new chemical structures and novel bioactivities unveiled by these methods are complementing the traditional bioassay-guided studies. However, as indicated by Glöckner and Joint [69], the success of the genome-based methods is largely dependent on the accuracy of bioinformatics analysis, and the bioinformatics tools are often the limiting factor. Researchers are now developing various bioinformatics tools such as ClustScan [70], NP.searcher [71], SBSPKS [72], to name a few, for the analysis of PKS or NRPS modular sequences and structure predictions. 

On the other hands new tools in the detection of natural products are continuously being developed to explore the chemical diversity of microbial natural products. The imaging mass spectrometry (IMS) tools were used to visualize the spatial distribution of secondary metabolites produced by marine cyanobacteria [73], providing information for dereplication and discovery of novel natural products. The PrISM (Proteomic Investigation of Secondary Metabolism) approach employs mass-spectrometry-based proteomics to localize expressed PKS or NRPS clusters, which resulted in the discovery of new NRPS gene cluster in *Bacillus* strain NK2018 [74]. Single-cell genomics approach was used to study the lifestyle of marine symbiotic bacteria, *Poribacteria* [75], and this strategy also holds promise in studying the biosynthetic potential of unculturable microorganisms in the near future. All these updated bioinformatics tools and detection tools can be combined to facilitate new marine natural products. 

**Figure 6:**
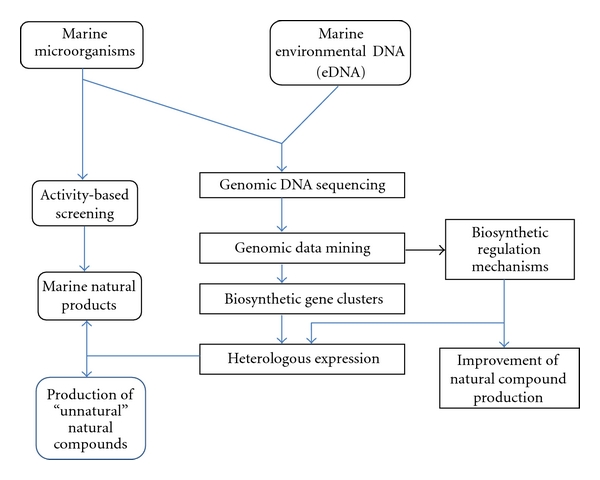
Combination of genome mining with classical activity-based screening for natural product discovery, generation, and hyperproduction.

The exploration of the diversity of marine microorganisms using the combined classical activity-based screening with the genome mining in both the culturable and unculturable microorganisms is presented in Figure 6. 

## 8. Conclusion and Prospects

Recent years have witnessed the exponentially increasing genome sequencing information, and genome mining analyses have revealed tremendous capability of marine microorganisms to produce active natural products as therapeutic agents. Studies on the biosynthetic machinery and regulatory mechanisms of natural product biosynthesis will greatly maximize the diversity exploration of marine natural products. Nowadays the diversity studies of natural products have been shift from pure chemical analysis to the combination of genetic manipulations and chemical synthesis, and the diversity of natural products can be further enlarged by genetic engineering of biosynthetic genes. Genomic research of marine microorganisms will have great impact on the discovery of novel natural products for medical treatments and will contribute to efficient drug discovery from marine environment.

## Figures and Tables

**Table 1:** Active natural products isolated from marine microorganisms.

Metabolites	Source	Activity	Reference	Clinical status
Soblidotin (TZT1027)	Synthetic derivative of dolastatin 10 isolated from marine cyanobacterium *Symploca* sp. VP642	Anticancer	[12]	Phase III
Plinabulin (NPI-2358)	Synthetic analog of halimide isolated from the marine fungus *Aspergillus* sp. CNC-139	Anticancer	[13]	Phase II
Bryostatin 1	Identified from bryozoan and also marine symbiotic bacteria *Candidatus Endobugula sertula *	Anticancer, Anti-Alzheimer	[14]	Phase I
Marizomib (salinosporamide A, NPI-0052)	Marine actinobacteria *Salinispora arenicola *	Anticancer	[15]	Phase I
Compound 4 (curacin A synthetic derivative)	Curacin A was isolated from the marine cyanobacterium *Lyngbya majuscula *	Anticancer	[16]	Preclinical
